# Unstructured kinetic models to simulate an arabinose switch that decouples cell growth from metabolite production

**DOI:** 10.1016/j.synbio.2020.07.003

**Published:** 2020-07-14

**Authors:** Harley Edwards, Peng Xu

**Affiliations:** Department of Chemical, Biochemical and Environmental Engineering, University of Maryland Baltimore County, Baltimore, MD, 21250, USA

**Keywords:** Metabolic switches, Biophysical models, Dynamic control, Metabolic engineering, Central carbon flux, Intelligent biomanufacturing, Green chemistry

## Abstract

Modeling synthetic gene circuits to implement dynamic flux balancing is crucial in teaching and exploring metabolic engineering strategies to repartition metabolic precursors and construct efficient microbial cell factories. Microbial fitness and production rates are often complex phenotypes that are governed by highly non-linear, multivariable functions which are intrinsically linked through carbon metabolism. The solution of such dynamic system can be difficult for synthetic biologists to visualize or conceptualize. Recently, researchers (Santala et al., Metab. Eng. Comm., 2018) have implemented an arabinose based genetic switch to dynamically partition the central carbon flux between cell growth and product formation. The autonomous switch allowed dynamic shift from arabinose-associated cell growth to acetate-associated product (wax ester) formation. This system clearly demonstrates the effectiveness of using a genetic switch to decouple cell growth from product formation in a one-pot bioreactor to minimize operational cost. Coupled with Michaelis-Menten kinetics, and Luedeking-Piret equations, we were able to reconstruct and analyze this metabolic switch *in silica* and achieved graphical solutions that qualitatively match with the experimental data. By assessing physiologically-accessible parameter space, we observed a wide range of dynamic behavior and examined the different limiting cases. Graphical solutions for this dynamic system can be viewed simultaneously and resolved in real time *via* buttons on the graphical user interface (GUI). Metabolic bottlenecks in the system can be accurately predicted by varying the respective rate constants. The GUI serves as a diagnosis toolkit to troubleshoot genetic circuits design constraints and as an interactive workflow of using this arabinose based genetic switch to dynamically control carbon flux, which may provide a valuable computational toolbox for metabolic engineers and synthetic biologists to simulate and understand complex genetic-metabolic system.

## Introduction

Microbes use a wide range of substrates with different energetic and redox states. These substrates may be assimilated to form biomass or final products. Examples of such substrates for microbial production include methane [[1], [2], [3]], acetate [[4], [5], [6]], or glycerol feedstocks [7,8]. Co-substrate utilization may serve to bypass some catabolic, energetically-costly steps, and facilitates more readily available anabolic precursors (i.e. acetyl-CoA) to synthesize final product [9], since the co-utilized substrate may form a metabolic bypass with less enzymatic steps, which otherwise could not be attained by the breakdown of the initial carbon source [10]. These feedstocks hold great promise in creating a sustainable, energy efficient commodity chemical production platform needed to supply a growing global population [11,12]. In an effort to further increase product yield, optimizing metabolic flux has long been accepted as a viable strategy by systems biologists when it comes to increasing carbon conversion along a metabolic pathway [[13], [14], [15]]. This type of flux balancing includes genetic knockouts to remove byproduct formation or competing metabolic steps, and genetic overexpression to increase rate-limiting metabolic steps [16]. Much of the effort concerns with the static regulation of metabolic flux without considering the hierarchically-organized regulatory architecture that is built into the cell metabolism [17,18].

Many simple regulatory genetic switches have been reconstructed and studied, for instance the genetic toggle switch [19], and the repressilator [20], which both demonstrate predictable gene expression pattern in a living system. Interdisciplinary knowledge of engineering design and synthetic biologic systems has paved the way toward development of plug-and-play genetic modules, whose behaviors exhibit a wide range of intriguingly dynamic behavior, including many logic gates [[21], [22], [23]], negative autoregulation [24,25], incoherent feedforward loops [26,27] and looped dual-level ON-OFF genetic circuit [[28], [29], [30]]. When coupled with quorum-sensing circuits, these genetic circuits may regulate gene expression at community or multiple-species level [[31], [32], [33], [34]]. Such synchronized gene expression is critical to eliminate genetics-associated metabolic heterogeneity [18]. As the complexity of modules increases, so does the difficulty due to a variety of factors like leaky expression due to trans-activity of metabolites, or via cross-talk of transcription factors as a result of non-orthogonality between two controlling modules [23,35,36].

**Fig. 1:**
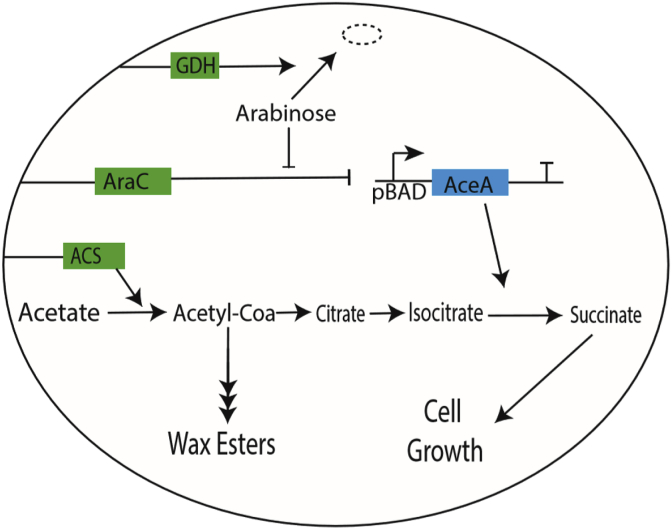
The above graphic depicts how arabinose is used as the signaling molecule to control the genetic switch from cell growth to product formation. Control scheme was redrawn by the work reported by Santala et al. [6]. Arabinose is the signal/induction molecule, and araC is the sensor/transducer, tightly regulating expression of *aceA* under the control of pBAD promoter. Exhaustion of arabinose limits the expression of the crucial enzyme, aceA, therefore shutting down cell growth, so carbon flow is diverted to wax ester production instead. This autonomous partition of carbon flux has been experimentally validated by the reported work.

In an elegant display of a metabolic switch being applied in a living system (*Acinetobacter baylyi*) grown on a simple carbon source (acetate), a team at Tampere University of Technology, Finland, was able to demonstrate dynamic control of a critical catabolic enzyme, such that the cells would automatically switch from cell growth to product formation, when the inducer arabinose was depleted [6]. The tight transcriptional control of the arabinose-responsive promoter could be easily tuned to increase carbon yield up to 3–4 fold. Specifically, this arabinose switch allows them to control the expression of critical enzymatic steps that are associated with cell growth and product formation. The arabinose inducible pBAD promoter was used to regulate the expression of isocitrate lyase, *aceA*, a key enzyme for the glyoxylate shunt pathway that replenishes precursors to the Krebs cycle that determine the fitness of cell growth. The input signal is arabinose, the sensor and transducer are a transcriptional repressor araC, the actuator is the *E. coli* native RNA polymerase, and the output signal is the expression of aceA. In the depletion of arabinose, araC tightly represses transcriptional activity of the pBAD promoter, thus shutting down glyoxylate activity and cell growth (Fig. 1). This metabolic switch effectively separates the cell growth phase from the product (wax ester) formation phase. Such growth-decoupled product formation in a one-pot bioreactor minimizes the use of expensive inducers (such as IPTG) or eliminates the use of a two-stage reactor, which may simplify the fermentation workflow and reduce the operational cost. In this work, we formulated an ODE system consisting of 12 equations to uncover the design constraints of such system. This computational framework may facilitate us to understand the dynamic control of gene expression and design precise behavior in a metabolic switch which further increases the cost-competitiveness of industrial fermentation.

### Computational method and formulation of system equations

The metabolic switch can be described mathematically on the basis of mass action, enzyme kinetics and transcriptional regulation models, specifically in Table 1. The cell growth followed Monod growth kinetics [37,38] based on succinate as a substrate only. Wax ester generation is dictated by a specific product formation rate which is comprised of a growth and non-growth associated component, governed by Luedeking-Piret model [39,40], but modeled with inhibitory effects due to substrate limitations. Since this was also modeled in batch configuration, the linear component of the specific product formation rate would cause the succinate to go to negative without considering the substrate inhibitory effect. The proteins responsible for the metabolism of acetyl-coA to isocitrate, were considered first order, elementary reactions that are constitutively coupled with cell biomass, and the enzyme consumption kinetics are only governed by a function of reactant concentration. Any other relevant reaction followed a traditional pseudo-steady state assumption to derive a Michaelis-Menton relationship between the enzyme and substrate.

**Table 1:** Equations used to define the arabinose based genetic switch for biomass and product decoupling.

Equation No.	Equations used in this work
1	$\frac{d\left[A\right]}{dt}=-\frac{k_{A1}\left[GDH\right]\;{\left[A\right]}^{n}}{K_{A1}^{n}+{\left[A\right]}^{n}}-\frac{k_{A2}\left[AraC\right]{\left[A\right]}^{m}}{K_{A2}^{m}+{\left[A\right]}^{m}}$
2	$\frac{d\left[GDH\right]}{dt}=\frac{\alpha_{g}\mu_{max}{\left[S\right]}^{w}}{K_{m}^{w}+{\left[S\right]}^{w}}X-\left[GDH\right]d_{g}$
3	$\frac{d\left[AraC\right]}{dt}=\frac{\alpha_{AraC}}{{\left(\frac{\left[A\right]}{K_{RA}}\right)}^{p}+1}-\left[AraC\right]d_{AraC}$
4	$\frac{d\left[ACS\right]}{dt}=\frac{\alpha_{ACS}\mu_{max}{\left[S\right]}^{w}\left[X\right]}{K_{m}^{w}+{\left[S\right]}^{w}}-\left[ACS\right]d_{ACS}$
5	$\frac{d\left[AceA\right]}{dt}=\frac{\alpha_{AceA}}{{\left(\frac{\left[AraC\right]}{K_{RAraC}}\right)}^{q}+1}-\left[AceA\right]d_{AceA}$
6	$\frac{d\left[Ac\right]}{dt}=-\frac{k_{Ac}\left[ACS\right]{\left[Ac\right]}^{r}}{K_{Ac}^{r}+{\left[Ac\right]}^{r}}$
7	$\frac{d\left[aCoA\right]}{dt}=\frac{k_{Ac}\left[ACS\right]{\left[Ac\right]}^{r}}{K_{Ac}^{r}+{\left[Ac\right]}^{r}}-\frac{\left(\frac{\alpha_{p}\mu_{max}{\left[S\right]}^{w}}{K_{m}^{w}+{\left[S\right]}^{w}}+\frac{\beta_{p}{\left[aCoA\right]}^{v}}{K_{PaCoA}^{v}+{\left[aCoA\right]}^{v}}\right)\left[X\right]}{Yps}-k_{aCoA}\left[aCoA\right]$
8	$\frac{d\left[WE\right]}{dt}=\left(\frac{\alpha_{p}\mu_{max}{\left[S\right]}^{w}}{K_{m}^{w}+{\left[S\right]}^{w}}+\frac{\beta_{p}{\left[aCoA\right]}^{v}}{K_{PaCoA}^{v}+{\left[aCoA\right]}^{v}}\right)\left[X\right]$
9	$\frac{d\left[C\right]}{dt}=\left[aCoA\right]k_{aCoA}-k_{c}\left[C\right]$
10	$\frac{d\left[Iso\right]}{dt}=k_{c}\left[C\right]-\frac{k_{Iso}\left[AceA\right]{\left[Iso\right]}^{s}}{K_{Iso}^{s}+{\left[Iso\right]}^{s}}$
11	$\frac{d\left[S\right]}{dt}=\frac{k_{Iso}\left[AceA\right]{\left[Iso\right]}^{s}}{K_{Iso}^{s}+{\left[Iso\right]}^{s}}-\frac{\mu_{max}{\left[S\right]}^{w}\left[X\right]}{\left(K_{m}^{w}+{\left[S\right]}^{w}\right)Yxs}$
12	$\frac{d\left[X\right]}{dt}=\frac{\mu_{max}{\left[S\right]}^{w}}{K_{m}^{w}+{\left[S\right]}^{w}}\left[X\right]-\left[X\right]d_{X}$
13	$\mu =\frac{1}{\left[X\right]}*\frac{d\left[X\right]}{dt}=\frac{\mu_{max}{\left[S\right]}^{w}}{K_{m}^{w}+{\left[S\right]}^{w}}$
14	${\text{q}}_{p}=\alpha_{p}\mu +\frac{\beta_{p}{\left[aCoA\right]}^{v}}{K_{PaCoA}^{v}+{\left[aCoA\right]}^{v}}$

The first 12 equations listed are mass balances which were input to MATLAB and solved via ODE23s. The last two equations, for specific growth rate and specific product formation rate, are there only listed for clarity. There are 12 independent equations, requiring 12 initial conditions, and 48 model parameters for a total of 60 variables. All numerical coefficient values can be found in the accompanying supplementary information file which contains the code. A graphical user interface was created to streamline the manipulation of this model, to compare the solution space under multiple variables at once, and to facilitate ease of demonstration and learning with this model. A detailed explanation of the biophysical parameters could be found in the symbol appendix section. Parameter values for these biophysical constants were estimated based on typical values of similar parameters found in literature [41].

### Computational methods

Matlab R2018b was used as the computational package on a Windows 10 professional operation system. The CPU processor is Intel Core i5-4300 with 1.90 GHz. The installed memory (RAM) is 4.0 GHz. Matlab symbolic language package coupled with LaTex was used to compile the equations (Table 1). ODE23s solver was used to simulate and predict the system behavior. Matlab plot function was used to output the solutions and graphs. Matlab codes will be shared upon request. Due to variations in native screen resolutions and default zoom, the GUI may not load into the correct position without adjustment.

### Parameters and initial conditions

**Fig. 2:**
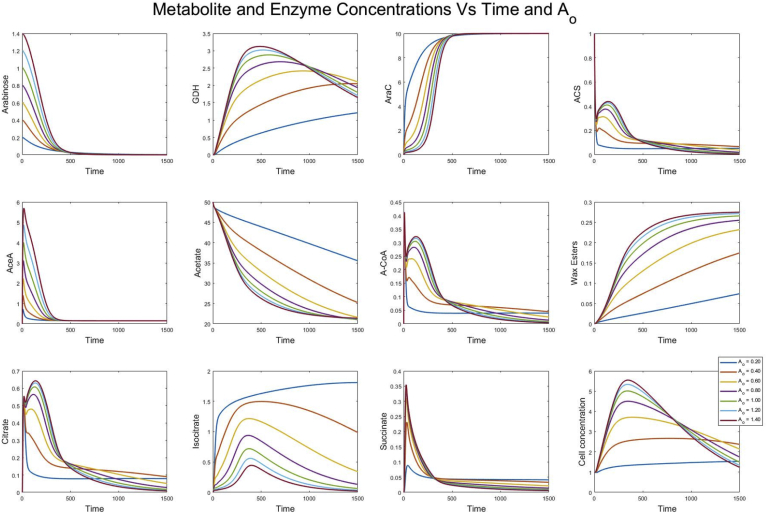
Investigating system dynamic behavior by varying initial concentration of arabinose. One can observe that with little arabinose, araC shoots up fast, aceA stays low, and very few cells grow. Increasing the initial arabinose causes a spike in cell biomass formed, a depression in araC, and an increase in aceA during the early time points. This allows more cells to grow in the beginning, draining the signaling molecule arabinose before wax ester could be synthesized. With arabinose ~0.4–0.6, one can observe a plateu of cells continuing to create wax esters. This represents high producing cells, creating high wax ester titers with lower cells, indicating a higher conversion yield of substrate to product with less wasted substrate or dead cells.

**Fig. 3:**
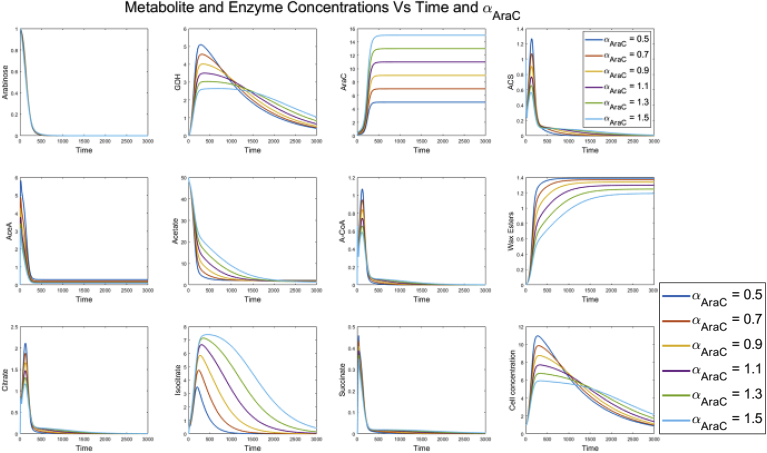
Effect of expression rate of araC on system dynamics. Low levels of araC expression effectively don't stop cells from growing and we see high levels of substrate consumed for cell growth. Since araC represses the expression of aceA that controls cell growth, high level of araC will lead to low level of succinate which limits cell growth from the control scheme described in Fig. 1.

Necessary parameters used to get the results in Fig. 2, Fig. 3 are below. The results directly supplemented in the supplementary Matlab code generate the results on the GUI screen. An addition was added to the supplementary files code so that it outputs the subplots of Fig. 2 as well as the GUI. Most of the parameters are taken form the Website BioNumbers (https://bionumbers.hms.harvard.edu/search.aspx) and the commonly-used biochemical engineering textbooks, written by Shuler and Kargi, Bioprocess Engineering [42].

A_o = 1, GDH_o = 0, AraC_o = 0, Acs_o = 1, AceA_o = 0, Ac_o = 50, aCoA_o = 0, WE_o = 0, C_o = 0, Iso_o = 0, S_o = 0, X_o = 1, k_1 = 0.1, K_1 = 40, n = 1, k_2 = 0.5, K_2 = 10, m = 2, alpha_g = 0.5, d_g = 0.001, alpha_A_r_a_C = 1,K_RA = 0.2, p = 2, d_AraC = 0.1, alpha_ACS = 1.7, d_ACS = 0.1, alpha_AceA = 1.5, K_RAraC = 0.1, q = 1, d_AceA = 0.1, k_Ac = 0.33,K_Ac = 10, r = 1, k_aCoA = 0.2, k_c = 0.1, k_Iso = 0.5, K_Iso = 20, s = 1, mu_max = 10, K_m = 200, w = 1, Yxs = 0.4, d_x = 0.001, alpha_p = 0.0000008, beta_p = 0.008, K_PaCoA = 5, v = 1, Yps = 0.1 … alpha_A_r_a_C = [.5:.2:1.5] …

## Results

### System dynamics by varying initial conditions of arabinose

**Fig. 4:**
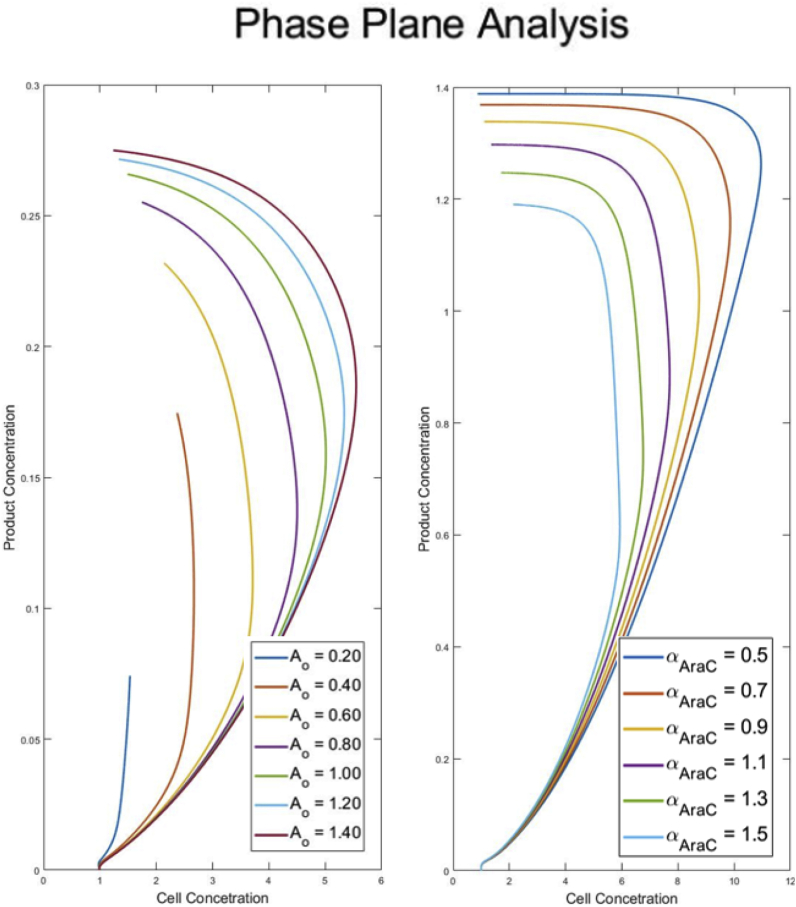
Phase plane analysis of both aforementioned scenarios, varying initial arabinose and varying expression strength of araC. We observed a positive correlation between biomass and product when the biomass is low, but a negative correlation between biomass and product when the biomass is high, which indicate the metabolic shift from biomass accumulation to wax ester buildup.

One of the important feature of this metabolic switch is to use arabinose as the signaling molecule to tune the cell growth rate, so that the substrate (acetate) consumption is diverted to product formation that leads to significant improvement in the pathway yield. By altering the initial concentration of arabinose (Fig. 1), the authors were able to achieve this tunability and effectively control the point at which carbon utilization would shift from cell growth to product formation. Our model recapitulates this tunability and clearly indicates there is decoupling of cell growth from product formation (Fig. 4).

In modeling this batch-process, we have set up a degradation constant *d* for each of the appropriate cellular components. In the case for cell growth, this term should be explained as ‘death rate’, while proteins have their own degradation rate, and the metabolites do not degrade or have a dilution term. Since the expression of GDH (glucose dehydrogenase) is coupled with biomass, we observed a similar pattern of GDH expression as compared to cell biomass (Fig. 1). Arabinose antagonizes the activity of AraC, that's why AraC in the system exhibits an opposite trajectory as compared to the arabinose in the system (Fig. 1). Similar negative correlations were found between the amount of AraC and aceA (Fig. 1), which is encoded by Eqn. 5 (Table 1). When the level of arabinose is depleted, our model predicts that increased acetate consumption leads to increased wax ester formation (Fig. 1). Interestingly, the amount of aceA (encoding isocitrate lyase), which dictates cell growth rate, initially increased quickly, but declined proportionally to the amount of arabinose left in the system. Therefore, the cell growth fitness, is positively correlated with the level of arabinose. As the cell grows, the accumulation of GDH increases the consumption rate for arabinose, therefore the critical enzyme aceA, and biomass, displays a decreasing pattern as the signaling molecule (arabinose) continues decreasing. These results indicate that our *in silico* models can precisely predict the switch from cell growth to product formation, depending solely on the level of arabinose in the system. These qualitatively dynamic behaviors were also experimentally validated by the authors.

### Effect of AraC expression rate on system dynamics

Since arabinose is antagonizing araC, the amount of active araC is negatively associated with arabinose (Eqn 3). We next investigated the system dynamics by varying expression levels of key proteins araC. The araC transcriptional regulator represses the expression of aceA under the control of pBAD, and the metabolic product of aceA (succinate) controls the glyoxylate shunt flux that determines cell growth rate. Experimentally, this kind of genetic manipulation could be achieved by changing gene copy number, promoter strength or *via* tuning the degradation rate constant *d*.

As expected, the level of GDH is strongly associated with the amount of biomass due to its constitutive expression (Fig. 3). AraC expression follows a Hill-type saturation kinetics as we increased the basal expression rate $\alpha_{AraC}$. The amount of aceA displays a ‘spike’ (single peak) pattern due to the strong repression of araC on the pBAD promoter which drives aceA expression (Fig. 3), indicating the system first favors cell growth but discourages cell growth after the spike. This single-peak pattern is also observed in the isocitrate, albeit the timing of the switching is significantly delayed. The final product, wax ester inverses the pattern of araC as we increase the expression rate of araC, indicating that low araC expression rates favors wax ester accumulation (Fig. 3). The correlation between biomass and product formation was investigated by a “phase-plane”, as described in Fig. 4. The phase-plane demonstrates a positive correlation between biomass and product when the biomass is low, but a negative correlation between biomass and product when the biomass is high. This phase-plane clearly indicates the metabolic shift from biomass accumulation to wax ester buildup.

### A GUI model to explore the parameter space

To explore the parameter space of the model, a GUI was developed so that this model could be analyzed more systematically. This made it significantly easier to tune the various parameters and determine whole system dynamic behavior. We discovered that some variables are order of magnitudes more sensitive than others, indicating that variables ranging relevantly from 0.01 to 1 may lead to similar dynamics as those parameters ranging from 1 to 100. Considering the fact that manual curation of the parameter space is very difficult and time consuming, this GUI may easily help us to find a biologically relevant window of initial parameters for all variables. With the scope for each variable included and a slider bar to change that variable in the GUI interface, we may explore the rich dynamic patterns of this dynamic system. The GUI makes the demonstration and interpretation of this model more intuitive, and explanatory than a traditional static graphical view.

**Fig. 5:**
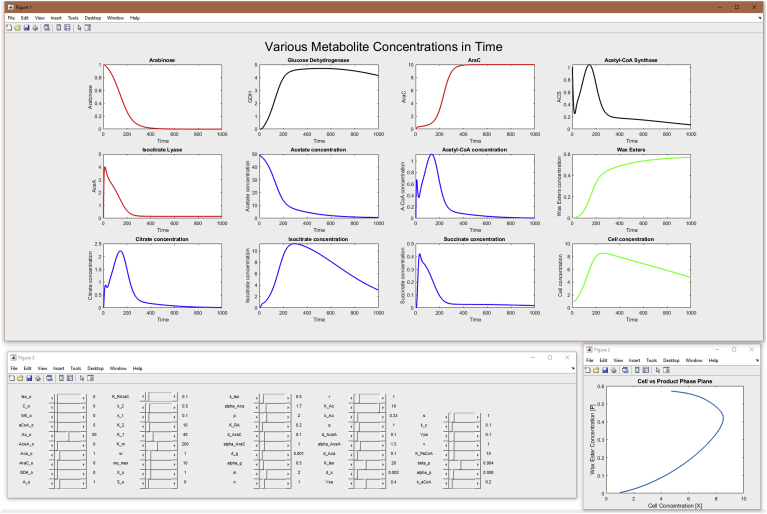
Demonstration of graphical user interface developed to dynamically manipulate the system in real time *via* slider bars to change parameters and initial conditions. Critical components of the arabinose switch are colored red, constitutive enzymes are black, metabolites are blue, and cell biomass and wax esters are in green. Phase plane is located to the bottom right.

The GUI is built using three separate instances of a new figure, with a listener and a callback function. The GUI figure on top represent solutions (Fig. 5), the bottom right represents a phase plane analysis, and the bottom left is the controller to change system variables and initial conditions (Fig. 5). The listener function monitors for controller buttons to be changed, and when the user interacts with one of the buttons, it triggers a function, which in this case is the callback function. The callback function rewrites the system variables to memory, using the new associated value from the controller, resolves the system of equations, and updates the plots with the associated solution. Every single variable of the system is accounted for and has a button, and as such, all must have their own listener. All listeners, however, point to the same callback function, which has to contain all variables in order to solve the system again. It is not to just replot the data or one would have many lines on the screen and have issues with visibility of scale. Instead, by making the callback function to change the line data, the plots appear to update in front of you in real time.

## Discussions

The phase plane analysis demonstrates two separate regimes of wax ester production pattern, for example, the initial linear regime carries the growth associated production term, but the verticality of these configurations represents the non-growth associated production of wax ester which is independent of cell growth. This simulation mathematically and qualitatively matches the results of the work reported by the researchers in Finland who found a four times increase in specific wax ester productivity by controlling carbon flux [6]. The key concept in the *in vivo* work is the tight regulation of critical catabolism through isocitrate lyase *via* the pBAD promoter, and linking that to the orthogonal arabinose inducer molecule arabinose. This tight regulation dictates the level of cell growth permitted and also governs when these cells stop growing and start producing the final product (wax ester). This elegant system was designed successfully to decouple cell growth and product formation, both of which are associated with carbon utilization. We reconstructed this genetic system *in silica* and demonstrate the effectiveness of this system to dynamically redistribute carbon flux. By working in *Acinetobacter baylyi*, there are two distinct advantages. They chose a host organism which natively relies on a shortened citric acid cycle, the glyoxylate shunt pathway, instead of the full TCA cycle. They also chose an organism whose native glucose dehydrogenase could not glycolytically degrade arabinose but would instead oxidize it into other inductively inactive pentoses. By intuitively choosing a host, and a feedstock, they effectively minimized the cloning work needed to be done to demonstrate success. They chose a product which very likely follows growth and non-growth associated production patterns. They chose a tight genetic switch, as well as a key enzyme required for growth as an output signal. In creating such a unique system, they have created an ideal case, which demonstrates a genetic toggle switch being implemented to control central carbon metabolism, effectively also controlling the carbon flux between growth and wax ester formation. Although this user interface might be helpful for explaining individual variables contributions toward system behavior, it is not perfect and is limited by initial conditions and the range of the variables set forth in the code. Each slider bar is a manually set range, and if someone decides to go outside of that range, this would require code manipulation. Another flaw is that in many of these complex nonlinear systems, imaginary numbers may give rise to unrealistic solutions which will not update in the graph correctly when the callback function is prompted to update the real number counterparts. This is because it would have to change the plot type, not just line values, in order to display complex answers. Some solutions also take a while to converge and cause the interface to lag before updating. The easiest way to troubleshoot what happened is to monitor the MATLAB command window, which will display an error if an imaginary number causes a bug or will loop in the solver indefinitely if it is not converging on a solution given that variable set. In both cases, re-running the code from the beginning will reestablish the GUI on the screen, in working order. This highlights another problem, that GUI is locked onto a local solution rather than the optimal solutions. One can move around within this solution set, but some variable region may lead to a highly stiff region or an imaginary solution set. Under this scenario, the GUI will not continue to function, and one could not probe variable values beyond that, unless they manually updated the code to change the GUI starting conditions and the range of the slider bars. The advantages of this extended type of graphical analysis include interactivity and a decreased level of involvement for manual input for small changes. Instead of changing one number in the code and replotting the graph, it is very easy to change, observe, and learn from the system by manipulating a slider bar on the user-interface. The disadvantage is primarily that the slider bars are range bound and stiff and unrealistic solutions cause the system to lag and need restarting.

Future work for this strain, or some congruent genetic configurations, could prove to be quite valuable. One might consider another model where arabinose is glycolytically consumable to the cell, a dual substrate model, where arabinose can be fed to the cell, and induce cell growth on acetate. Once arabinose was gone, along with all the glycolytic catabolites from supporting growth, one would hypothesize the phenomena observed in this paper would take over. Another interesting case could be growing this strain in CSTR conditions. One might think of alternatively pulse-feeding substrate and arabinose for highest yields. This is only one application in one host organism, whereas this is hypothetically possible in any microbial workhorse genetically tractable enough to knockout the greater TCA cycle in favor of the glyoxylate shunt. Researchers may have the flexibility to use a different inducer/actuator module with strain specific orthogonality, and other synthetic biology-based logic gates, genetically-encoded biosensors [43], [44] and genetic switches may also be integrated to improve the system robustness and predictability.

## Conclusions

By simulating this arabinose-based genetic switch, we demonstrate our model could effectively recapitulate the dynamics of the metabolic shift from cell growth to product formation, solely dependent on the exogenously added inexpensive carbons (arabinose). We also demonstrate that a graphical representation of this model can be immensely helpful in analyzing and understanding the dynamic behavior of gene circuits. This model system in *A. baylyi* represents a potentially valuable discovery in terms of maximizing carbon conversion and product yield from substrates, and minimizing bioburden from gene overexpression or host cell protein associated with certain hosts. The tight regulation of isocitrate lyase by the arabinose inducible pBAD promoter offers an ideal control scheme to tightly improve cell productivity though central carbon metabolism. The simplicity of the proposed models is sufficient to describe genetic circuits dynamics, yet extendable to understand the dynamic carbon flux balancing in various organisms. This system may hold incredible promise to facilitate learning and engaging students with these complex, often abstract, and intertwined ideas of microbial growth, genetic circuits, enzyme kinetics, and coupled differential equations.

## Declaration of competing interest

The author declares no conflicts of interests.

## Appendix Symbols and variables used in this work

[A]: Arabinose Concentration
[GDH]: Glucose Dehydrogenase Concentration
[AraC]: Arabinose Operon Regulatory Protein
[ACS]: Acetyl-CoA Synthase Concentration
[AceA]: Isocitrate Lyase Concentration
[Ac]: Acetate Concentration
[aCoA]: Acetyl-CoA Concentration
[WE]: Wax Ester Concentration
[C]: Citrate Concentration
[Iso]: Isocitrate Concentration
[S]: Succinate Concentration
[X]: Cell Concentration
Y_X/S_: Biomass per Substrate Yield Coefficient
Y_P/S_: Product per Substrate Yield Coefficient
α_i_: Expression rate of protein i
β_i_: Non-Growth Associated Product Formation Rate
n,m,p,q,r,s,v,w: Hill Coefficients
k_i,j_: Rate Constants for reactant I, reaction j. If there's no j, that's indicative of one reaction
K_i,j_: Equilibrium Constants for reactant I, reaction j
K_R,i_: Repression Equilibrium Constant Due to Repression from i
K_P,I_: Equilibrium Constant for Products and Reactant i
d_i_: Degradation of species i
β_X_: Cell death/loss rate
