# Supplementary material for: Unstructured kinetic models to simulate an arabinose switch that decouples cell growth from metabolite production
Source: Synth Syst Biotechnol. 2020 Jul 14;5(3):222–9. doi: 10.1016/j.synbio.2020.07.003 (PMC7364165; doi:10.1016/j.synbio.2020.07.003)
Supplement: Multimedia component 1 [file mmc1.pdf]

Unstructured kinetic models to simulate an arabinose switch that decouples cell growth from metabolite production

Harley Edwards, Peng Xu*

Supplementary files for Arabinose Switch GUI

Arabinose Multiplot Gui 1

Run ODE 6

Make Plots 6

Make Gui Controller 8

Function to rewrite old variables, resolve ODEs and update plots. 17

Arabinose Multiplot Gui

Harley Edwards

```
function Arabinos_Gui
```

```
clear all; clc
hold all


A_o = 1;
GDH_o = 0;
AraC_o = 0;
Acs_o = 1;
AceA_o = 0;
Ac_o = 50;
aCoA_o = 0;
WE_o = 0;
C_o = 0;
Iso_o = 0;
S_o = 0;
X_o = 1;
k_1 = 0.1;
K_1 = 40;
n = 1;
k_2 = 0.5;
K_2 = 10;
m = 2;
alpha_g = 0.5;
d_g = 0.001;
alpha_A_r_a_C = 1.5;
```

```
K_RA = 0.2;
p = 2;
d_AraC = 0.1;
alpha_ACS = 1.7;
d_ACS = 0.1;
alpha_AceA = 1.5;
K_RAraC = 0.1;
q = 1;
d_AceA = 0.1;
k_Ac = 0.33;
K_Ac = 10;
r = 1;
k_aCoA = .2;
k_c=0.1;
k_Iso = 0.5;
K_Iso = 20;
s = 1;
mu_max = 10;
K_m = 200;
w = 1;
Yxs=0.4;
d_x = 0.001;
alpha_p = .0000008;
beta_p = 0.008;
K_PaCoA = 5;
v = 1;
Yps = 0.1;

for alpha_A_r_a_C = [.2:.2:1.5]
tspan = [1:1:3000];
initial = [A_o GDH_o AraC_o Acs_o AceA_o Ac_o aCoA_o WE_o C_o Iso_o S_o X_o];
options = ['NonNegative', 1];

[T,Y]=ode23s(@(t,x) Arabinose_Gui_Functions(t,x, k_1, K_1, n, k_2, K_2, m, alpha_g, d_g,
alpha_A_r_a_C, K_RA, p, d_AraC, alpha_ACS, d_ACS,alpha_AceA, K_RAraC, q, d_AceA, k_Ac, K_Ac, r,
k_aCoA, k_c, k_Iso, K_Iso, s, mu_max, K_m, w,Yxs, d_x, alpha_p, beta_p, K_PaCoA, v, Yps),tspan,
initial, options);

figure(1)
subplot(3,4,1)
subtit = sgtitle('Metabolite and Enzyme Concentrations Vs Time and \alpha_A_r_a_C')
subtit.FontSize = 30

subplot(3,4,1)
%figure(1)
plot(T, Y(:,1), 'Linewidth', 2, 'DisplayName',sprintf('\alpha_A_r_a_C = %0.2f',alpha_A_r_a_C))
xlabel('Time', 'FontSize', 15);
ylabel('Arabinose', 'FontSize', 15);
%title('Arabinose');
hold on

subplot(3, 4, 2)
%figure(2)
```

```
plot(T, Y(:,2), 'Linewidth', 2,'DisplayName',sprintf('\alpha_A_r_a_C = %0.2f',alpha_A_r_a_C))
xlabel('Time', 'FontSize', 15);
ylabel('GDH', 'FontSize', 15);
%title('Glucose Dehydrogenase');
hold on

subplot(3, 4, 3)
%figure(3)
plot(T, Y(:,3), 'Linewidth', 2,'DisplayName',sprintf('\alpha_A_r_a_C = %0.2f',alpha_A_r_a_C))
xlabel('Time', 'FontSize', 15);
ylabel('AraC', 'FontSize', 15);
%title('AraC');
hold on

subplot(3, 4, 4)
%figure(4)
plot(T, Y(:,4), 'Linewidth', 2,'DisplayName',sprintf('\alpha_A_r_a_C = %0.2f',alpha_A_r_a_C))
xlabel('Time', 'FontSize', 15);
ylabel('ACS', 'FontSize', 15);
%title('Acetyl-CoA Synthase');
hold on

subplot(3, 4, 5)
%figure(5)
plot(T, Y(:,5), 'Linewidth', 2,'DisplayName',sprintf('\alpha_A_r_a_C = %0.2f',alpha_A_r_a_C))
xlabel('Time', 'FontSize', 15);
ylabel('AceA', 'FontSize', 15)
%title('Isocitrate Lyase');
hold on

subplot(3, 4, 6)
%figure(6)
plot(T, Y(:,6), 'Linewidth', 2,'DisplayName',sprintf('\alpha_A_r_a_C = %0.2f',alpha_A_r_a_C))
xlabel('Time', 'FontSize', 15);
ylabel('Acetate', 'FontSize', 15)
%title('Acetate concentration');
hold on

subplot(3, 4, 7)
%figure(7)
plot(T, Y(:,7), 'Linewidth', 2,'DisplayName',sprintf('\alpha_A_r_a_C = %0.2f',alpha_A_r_a_C))
xlabel('Time', 'FontSize', 15);
ylabel('A-CoA', 'FontSize', 15);
%title('Acetyl-CoA concentration');
hold on

subplot(3, 4, 8)
%figure(8)
plot(T, Y(:,8), 'Linewidth', 2,'DisplayName',sprintf('\alpha_A_r_a_C = %0.2f',alpha_A_r_a_C))
xlabel('Time', 'FontSize', 15);
ylabel('Wax Esters', 'FontSize', 15)
%title('Wax Esters');
hold on
```

```
subplot(3, 4, 9)
%figure(9)
plot(T, Y(:,9), 'Linewidth', 2,'DisplayName',sprintf('\alpha_A_r_a_C = %0.2f',alpha_A_r_a_C))
xlabel('Time', 'FontSize', 15);
ylabel('Citrate', 'FontSize', 15)
%title('Citrate concentration');
hold on

subplot(3, 4, 10)
%figure(10)
plot(T, Y(:,10), 'Linewidth', 2,'DisplayName',sprintf('\alpha_A_r_a_C = %0.2f',alpha_A_r_a_C))
xlabel('Time', 'FontSize', 15);
ylabel('Isocitrate', 'FontSize', 15)
%title('Isocitrate concentration');
hold on

subplot(3, 4, 11)
%figure(11)
plot(T, Y(:,11), 'Linewidth', 2,'DisplayName',sprintf('\alpha_A_r_a_C = %0.2f',alpha_A_r_a_C))
xlabel('Time', 'FontSize', 15);
ylabel('Succinate', 'FontSize', 15)
%title('Succinate concentration');
hold on

subplot(3, 4, 12)
%figure(12)
plot(T, Y(:,12), 'Linewidth', 2,'DisplayName',sprintf('\alpha_A_r_a_C = %0.2f',alpha_A_r_a_C))
xlabel('Time', 'FontSize', 15);
ylabel('Cell concentration', 'FontSize', 15)
%title('Cell concentration');
hold on

figure(2)
subplot(1,3,1)
sgtitle('Phase Plane Analysis', 'FontSize', 20)
plot(Y(:,12),Y(:,8), 'Linewidth', 2, 'DisplayName',sprintf('\alpha_A_r_a_C =
%0.2f',alpha_A_r_a_C))
xlabel('Cell Concetration', 'FontSize', 15);
ylabel('Product Concentration', 'FontSize', 15)
hold on

subplot(1,3,2)
plot(Y(:,6),Y(:,12), 'Linewidth', 2, 'DisplayName',sprintf('\alpha_A_r_a_C =
%0.2f',alpha_A_r_a_C))
ylabel('Cell Concetration', 'FontSize', 15);
xlabel('Acetate Concentration', 'FontSize', 15)
hold on

subplot(1,3,3)
plot(Y(:,6),Y(:,8), 'Linewidth', 2, 'DisplayName',sprintf('\alpha_A_r_a_C =
%0.2f',alpha_A_r_a_C))
ylabel('Product Concentration', 'FontSize', 15);
```

```
xlabel('Acetate Concentration', 'FontSize', 15)
hold on


end
legend({'\alpha_A_r_a_C = 0.5','\alpha_A_r_a_C = 0.7','\alpha_A_r_a_C = 0.9','\alpha_A_r_a_C =
1.1','\alpha_A_r_a_C = 1.3','\alpha_A_r_a_C = 1.5'}, 'location','best', 'FontSize', 15,
'interpreter', 'tex')


clear all

% Set some parameters

t2 = 1000

A_o = 1;
GDH_o = 0;
AraC_o = 0;
Acs_o = 1;
AceA_o = 0;
Ac_o = 50;
aCoA_o = 0;
WE_o = 0;
C_o = 0;
Iso_o = 0;
S_o = 0;
X_o = 1;

k_1 = 0.1;
K_1 = 40;
n = 1;

k_2 = 0.5;
K_2 = 10;
m = 2;

alpha_g = 0.5;
d_g = 0.001;

beta_AraC = 1;
K_RA = 0.2;
p = 2;
d_AraC = 0.1;

alpha_ACS = 1.7;
d_ACS = 0.1;

beta_AceA = 1.5;
K_RAraC = 0.1;
q = 1;
d_AceA = 0.1;
```

```
k_Ac = 0.2;
K_Ac = 10;
r = 1;

k_aCoA = .2;
k_c=0.1;
k_Iso = 0.5;
K_Iso = 5;
s = 1;
mu_max = 10;
K_m = 200;
w = 1;
Yxs=0.4;
beta_x = 0.002;
alpha_p = .008;
beta_p = 0.004;
K_PaCoA = 10;
v = 1;
Yps = 0.1;

Params = [k_1 K_1 n k_2 K_2 m alpha_g d_g beta_AraC K_RAraC...
    p d_AraC alpha_ACS d_ACS beta_AceA K_RA q d_AceA k_Ac K_Ac...
    r k_aCoA k_c k_Iso K_Iso s mu_max K_m w Yxs beta_x alpha_p...
    beta_p K_PaCoA v Yps]


tspan = [1:1:t2];
initial = [A_o GDH_o AraC_o ACs_o AceA_o Ac_o aCoA_o WE_o C_o Iso_o S_o X_o];
options = ['NonNegative', 1];
```

Run ODE

```
[T,Y]=ode23s(@(t,x) Arabinose_Gui_Functions(t,x, k_1, K_1, n, k_2, K_2, m, alpha_g, d_g,
beta_AraC, K_RA, p, d_AraC, alpha_ACS, d_ACS,beta_AceA, K_RAraC, q, d_AceA, k_Ac, K_Ac, r,
k_aCoA, k_c, k_Iso, K_Iso, s, mu_max, K_m, w,Yxs, beta_x, alpha_p, beta_p, K_PaCoA, v,
Yps),tspan, initial, options);
```

Make Plots

```
Multiplot = figure('Position', [50, 550, 2000, 800])

subplot(3,4,1)
LineA = plot(T,Y(:,1),'r','LineWidth',2);
xlabel('time');
ylabel('Arabinose');
title('Arabinose vs time');


subplot(3, 4, 2)
LineGDH = plot(T,Y(:,2), 'k','LineWidth',2)
xlabel('time');
```

```
ylabel('GDH');
title('Glucose Dehydrogenase vs time');


subplot(3, 4, 3)
LineAraC = plot(T, Y(:,3), 'r','LineWidth',2)
xlabel('time');
ylabel('AraC');
title('AraC vs time');


subplot(3, 4, 4)
LineACS = plot(T, Y(:,4), 'k','LineWidth',2)
xlabel('time');
ylabel('ACS');
title('Acetyl-CoA Synthase vs time');


subplot(3, 4, 5)
LineAceA = plot(T, Y(:,5), 'r','LineWidth',2)
xlabel('time');
ylabel('AceA')
title('Isocitrate Lyase vs time');


subplot(3, 4, 6)
LineAc=plot(T, Y(:,6), 'b','LineWidth',2)
xlabel('time');
ylabel('Acetate concentration')
title('Acetate concentration vs time');


subplot(3, 4, 7)
LineaCoA=plot(T, Y(:,7), 'b','LineWidth',2)
xlabel('time');
ylabel('A-CoA concentration');
title('Acetyl-CoA concentration vs time');


subplot(3, 4, 8)
LineWE=plot(T, Y(:,8), 'g','LineWidth',2)
xlabel('time');
ylabel('Wax Esters concentration')
title('Wax Esters vs time');


subplot(3, 4, 9)
LineC=plot(T, Y(:,9), 'b','LineWidth',2)
xlabel('time');
ylabel('Citrate concentration')
title('Citrate concentration vs time');
```

```
subplot(3, 4, 10)
LineIso=plot(T, Y(:,10), 'b','LineWidth',2)
xlabel('time');
ylabel('Isocitrate concentration')
title('Isocitrate concentration vs time');


subplot(3, 4, 11)
LineS=plot(T, Y(:,11), 'b','LineWidth',2)
xlabel('time');
ylabel('Succinate concentration')
title('Succinate concentration vs time');


subplot(3, 4, 12)
LineX=plot(T, Y(:,12), 'g','LineWidth',2)
xlabel('time');
ylabel('Cell concentration')
title('Cell concentration vs time');

figure('Position', [1600, 100, 500, 400])
PhasePlane = plot(Y(:,12),Y(:,8),'LineWidth',2);
xlabel('Cell Concentration [X]');
ylabel('Wax Ester Concentration [P]')
title('Cell vs Product Phase Plane');
```

Make Gui Controller

```
Controller = figure('Position', [50, 200, 1500, 300])

% Each variable has a slider, a title, and a value in that order.

%First Collumn

% A_o
Con_A_o = uicontrol('Parent',Controller,'Style','slider','Position',[110, 25, 100, 25],...
              'value',A_o, 'min',0, 'max',10, 'SliderStep',[0.01 0.10]);
bgcolor = Controller.Color;
Con_A_o_Title= uicontrol('Parent',Controller,'Style','text','Position',[1,25,100,25],...
    'String','A_o','BackgroundColor',bgcolor);
Con_A_o_Value= uicontrol('Parent',Controller,'Style','text','Position',[210,25,40,25],...
    'String',num2str(A_o),'BackgroundColor',bgcolor);
% GDH_o
Con_GDH_o = uicontrol('Parent',Controller,'Style','slider','Position',[110, 50, 100, 25],...
              'value',GDH_o, 'min',0, 'max',10, 'SliderStep',[0.01 0.10]);
bgcolor = Controller.Color;
Con_GDH_o_Title= uicontrol('Parent',Controller,'Style','text','Position',[1,50,100,25],...
    'String','GDH_o','BackgroundColor',bgcolor);
Con_GDH_o_Value= uicontrol('Parent',Controller,'Style','text','Position',[210,50,40,25],...
    'String',num2str(GDH_o),'BackgroundColor',bgcolor);
% AraC_o
Con_AraC_o = uicontrol('Parent',Controller,'Style','slider','Position',[110, 75, 100, 25],...
```

```
                'value',AraC_o, 'min',0, 'max',10, 'SliderStep',[0.01 0.10]);
bgcolor = Controller.Color;
Con_AraC_o_Title= uicontrol('Parent',Controller,'Style','text','Position',[1,75,100,25],...
    'String','AraC_o','BackgroundColor',bgcolor);
Con_AraC_o_Value= uicontrol('Parent',Controller,'Style','text','Position',[210,75,40,25],...
    'String',num2str(AraC_o),'BackgroundColor',bgcolor);
% Acs_o
Con_Acs_o = uicontrol('Parent',Controller,'Style','slider','Position',[110, 100, 100, 25],...
                'value',Acs_o, 'min',0, 'max',10, 'SliderStep',[0.01 0.10]);
bgcolor = Controller.Color;
Con_Acs_o_Title= uicontrol('Parent',Controller,'Style','text','Position',[1,100,100,25],...
    'String','Acs_o','BackgroundColor',bgcolor);
Con_Acs_o_Value= uicontrol('Parent',Controller,'Style','text','Position',[210,100,40,25],...
    'String',num2str(Acs_o),'BackgroundColor',bgcolor);
% AceA_o
Con_AceA_o = uicontrol('Parent',Controller,'Style','slider','Position',[110, 125, 100, 25],...
                'value',AceA_o, 'min',0, 'max',10, 'SliderStep',[0.01 0.10]);
bgcolor = Controller.Color;
Con_AceA_o_Title= uicontrol('Parent',Controller,'Style','text','Position',[1,125,100,25],...
    'String','AceA_o','BackgroundColor',bgcolor);
Con_AceA_o_Value= uicontrol('Parent',Controller,'Style','text','Position',[210,125,40,25],...
    'String',num2str(AceA_o),'BackgroundColor',bgcolor);
% Ac_o
Con_Ac_o = uicontrol('Parent',Controller,'Style','slider','Position',[110, 150, 100, 25],...
                'value',Ac_o, 'min',0, 'max',100, 'SliderStep',[0.01 0.10]);
bgcolor = Controller.Color;
Con_Ac_o_Title= uicontrol('Parent',Controller,'Style','text','Position',[1,150,100,25],...
    'String','Ac_o','BackgroundColor',bgcolor);
Con_Ac_o_Value= uicontrol('Parent',Controller,'Style','text','Position',[210,150,40,25],...
    'String',num2str(Ac_o),'BackgroundColor',bgcolor);
% aCoA_o
Con_aCoA_o = uicontrol('Parent',Controller,'Style','slider','Position',[110, 175, 100, 25],...
                'value',aCoA_o, 'min',0, 'max',10, 'SliderStep',[0.01 0.10]);
bgcolor = Controller.Color;
Con_aCoA_o_Title= uicontrol('Parent',Controller,'Style','text','Position',[1,175,100,25],...
    'String','aCoA_o','BackgroundColor',bgcolor);
Con_aCoA_o_Value= uicontrol('Parent',Controller,'Style','text','Position',[210,175,40,25],...
    'String',num2str(aCoA_o),'BackgroundColor',bgcolor);
% WE_o
Con_WE_o = uicontrol('Parent',Controller,'Style','slider','Position',[110, 200, 100, 25],...
                'value',WE_o, 'min',0, 'max',10, 'SliderStep',[0.01 0.10]);
bgcolor = Controller.Color;
Con_WE_o_Title= uicontrol('Parent',Controller,'Style','text','Position',[1,200,100,25],...
    'String','WE_o','BackgroundColor',bgcolor);
Con_WE_o_Value= uicontrol('Parent',Controller,'Style','text','Position',[210,200,40,25],...
    'String',num2str(WE_o),'BackgroundColor',bgcolor);
% C_o
Con_C_o = uicontrol('Parent',Controller,'Style','slider','Position',[110, 225, 100, 25],...
                'value',C_o, 'min',0, 'max',10, 'SliderStep',[0.01 0.10]);
bgcolor = Controller.Color;
Con_C_o_Title= uicontrol('Parent',Controller,'Style','text','Position',[1,225,100,25],...
    'String','C_o','BackgroundColor',bgcolor);
Con_C_o_Value= uicontrol('Parent',Controller,'Style','text','Position',[210,225,40,25],...
```

```
    'String',num2str(C_o),'BackgroundColor',bgcolor);
% Iso_o
Con_Iso_o = uicontrol('Parent',Controller,'Style','slider','Position',[110, 250, 100, 25],...
              'value',Iso_o, 'min',0, 'max',10, 'SliderStep',[0.01 0.10]);
bgcolor = Controller.Color;
Con_Iso_o_Title= uicontrol('Parent',Controller,'Style','text','Position',[1,250,100,25],...
    'String','Iso_o','BackgroundColor',bgcolor);
Con_Iso_o_Value= uicontrol('Parent',Controller,'Style','text','Position',[210,250,40,25],...
    'String',num2str(Iso_o),'BackgroundColor',bgcolor);




% Second Collumn
% S_o
Con_S_o = uicontrol('Parent',Controller,'Style','slider','Position',[350, 25, 100, 25],...
              'value',S_o, 'min',0, 'max',10, 'SliderStep',[0.01 0.10]);
bgcolor = Controller.Color;
Con_S_o_Title= uicontrol('Parent',Controller,'Style','text','Position',[250,25,100,25],...
    'String','S_o','BackgroundColor',bgcolor);
Con_S_o_Value= uicontrol('Parent',Controller,'Style','text','Position',[450,25,40,25],...
    'String',num2str(S_o),'BackgroundColor',bgcolor);
% X_o
Con_X_o = uicontrol('Parent',Controller,'Style','slider','Position',[350, 50, 100, 25],...
              'value',X_o, 'min',0, 'max',10, 'SliderStep',[0.01 0.10]);
bgcolor = Controller.Color;
Con_X_o_Title= uicontrol('Parent',Controller,'Style','text','Position',[250,50,100,25],...
    'String','X_o','BackgroundColor',bgcolor);
Con_X_o_Value= uicontrol('Parent',Controller,'Style','text','Position',[450,50,40,25],...
    'String',num2str(X_o),'BackgroundColor',bgcolor);

% mu_max
Con_mu_max = uicontrol('Parent',Controller,'Style','slider','Position',[350, 75, 100, 25],...
              'value',mu_max, 'min',0, 'max',100, 'SliderStep',[0.01 0.10]);
bgcolor = Controller.Color;
Con_mu_max_Title= uicontrol('Parent',Controller,'Style','text','Position',[250,75,100,25],...
    'String','mu_max','BackgroundColor',bgcolor);
Con_mu_max_Value= uicontrol('Parent',Controller,'Style','text','Position',[450,75,40,25],...
    'String',num2str(mu_max),'BackgroundColor',bgcolor);

% w
Con_w = uicontrol('Parent',Controller,'Style','slider','Position',[350, 100, 100, 25],...
              'value',w, 'min',0, 'max',10, 'SliderStep',[0.05 0.10]);
bgcolor = Controller.Color;
Con_w_Title= uicontrol('Parent',Controller,'Style','text','Position',[250,100,100,25],...
    'String','w','BackgroundColor',bgcolor);
Con_w_Value= uicontrol('Parent',Controller,'Style','text','Position',[450,100,40,25],...
    'String',num2str(w),'BackgroundColor',bgcolor);

% K_m
Con_K_m = uicontrol('Parent',Controller,'Style','slider','Position',[350, 125, 100, 25],...
              'value',K_m, 'min',0, 'max',500, 'SliderStep',[0.001 0.10]);
bgcolor = Controller.Color;
```

```
Con_K_m_Title= uicontrol('Parent',Controller,'Style','text','Position',[250,125,100,25],...
    'String','K_m','BackgroundColor',bgcolor);
Con_K_m_Value= uicontrol('Parent',Controller,'Style','text','Position',[450, 125,40,25],...
    'String',num2str(K_m),'BackgroundColor',bgcolor);

% K_1
Con_K_1 = uicontrol('Parent',Controller,'Style','slider','Position',[350, 150, 100, 25],...
              'value',K_1, 'min',0, 'max',200, 'SliderStep',[0.01 0.10]);
bgcolor = Controller.Color;
Con_K_1_Title= uicontrol('Parent',Controller,'Style','text','Position',[250,150,100,25],...
    'String','K_1','BackgroundColor',bgcolor);
Con_K_1_Value= uicontrol('Parent',Controller,'Style','text','Position',[450,150,40,25],...
    'String',num2str(K_1),'BackgroundColor',bgcolor);

% K_2
Con_K_2 = uicontrol('Parent',Controller,'Style','slider','Position',[350, 175, 100, 25],...
              'value',K_2, 'min',0, 'max',200, 'SliderStep',[0.01 0.10]);
bgcolor = Controller.Color;
Con_K_2_Title= uicontrol('Parent',Controller,'Style','text','Position',[250,175,100,25],...
    'String','K_2','BackgroundColor',bgcolor);
Con_K_2_Value= uicontrol('Parent',Controller,'Style','text','Position',[450,175,40,25],...
    'String',num2str(K_2),'BackgroundColor',bgcolor);

% k_1
Con_k_1 = uicontrol('Parent',Controller,'Style','slider','Position',[350, 200, 100, 25],...
              'value',k_1, 'min',0, 'max',10, 'SliderStep',[0.01 0.10]);
bgcolor = Controller.Color;
Con_k_1_Title= uicontrol('Parent',Controller,'Style','text','Position',[250,200,100,25],...
    'String','k_1','BackgroundColor',bgcolor);
Con_k_1_Value= uicontrol('Parent',Controller,'Style','text','Position',[450,200,40,25],...
    'String',num2str(k_1),'BackgroundColor',bgcolor);

% k_2
Con_k_2 = uicontrol('Parent',Controller,'Style','slider','Position',[350, 225, 100, 25],...
              'value',k_2, 'min',0, 'max',10, 'SliderStep',[0.01 0.10]);
bgcolor = Controller.Color;
Con_k_2_Title= uicontrol('Parent',Controller,'Style','text','Position',[250,225,100,25],...
    'String','k_2','BackgroundColor',bgcolor);
Con_k_2_Value= uicontrol('Parent',Controller,'Style','text','Position',[450,225,40,25],...
    'String',num2str(k_2),'BackgroundColor',bgcolor);
% K_RAraC
Con_K_RAraC = uicontrol('Parent',Controller,'Style','slider','Position',[350, 255, 100, 25],...
              'value',K_RAraC, 'min',0, 'max',10, 'SliderStep',[0.01 0.10]);
bgcolor = Controller.Color;
Con_K_RAceA_Title= uicontrol('Parent',Controller,'Style','text','Position',[250,250,100,25],...
    'String','K_RAraC','BackgroundColor',bgcolor);
Con_K_RAraC_Value= uicontrol('Parent',Controller,'Style','text','Position',[450,250,40,25],...
    'String',num2str(K_RAraC),'BackgroundColor',bgcolor);




%Third Collumn
```

```
% n
Con_n = uicontrol('Parent',Controller,'Style','slider','Position',[650, 25, 100, 25],...
              'value',n, 'min',0, 'max',10, 'SliderStep',[0.05 0.10]);
bgcolor = Controller.Color;
Con_n_Title= uicontrol('Parent',Controller,'Style','text','Position',[550,25,100,25],...
    'String','n','BackgroundColor',bgcolor);
Con_n_Value= uicontrol('Parent',Controller,'Style','text','Position',[750,25,40,25],...
    'String',num2str(n),'BackgroundColor',bgcolor);
% m
Con_m = uicontrol('Parent',Controller,'Style','slider','Position',[650, 50, 100, 25],...
              'value',m, 'min',0, 'max',10, 'SliderStep',[0.05 0.10]);
bgcolor = Controller.Color;
Con_m_Title= uicontrol('Parent',Controller,'Style','text','Position',[550,50,100,25],...
    'String','m','BackgroundColor',bgcolor);
Con_m_Value= uicontrol('Parent',Controller,'Style','text','Position',[750,50,40,25],...
    'String',num2str(m),'BackgroundColor',bgcolor);
% alpha_g
Con_alpha_g = uicontrol('Parent',Controller,'Style','slider','Position',[650, 75, 100, 25],...
              'value',alpha_g, 'min',0, 'max',10, 'SliderStep',[0.01 0.10]);
bgcolor = Controller.Color;
Con_alpha_g_Title= uicontrol('Parent',Controller,'Style','text','Position',[550,75,100,25],...
    'String','alpha_g','BackgroundColor',bgcolor);
Con_alpha_g_Value= uicontrol('Parent',Controller,'Style','text','Position',[750,75,40,25],...
    'String',num2str(alpha_g),'BackgroundColor',bgcolor);
% d_g
Con_d_g = uicontrol('Parent',Controller,'Style','slider','Position',[650, 100, 100, 25],...
              'value',d_g, 'min',0, 'max',1, 'SliderStep',[0.001 0.10]);
bgcolor = Controller.Color;
Con_d_g_Title= uicontrol('Parent',Controller,'Style','text','Position',[550,100,100,25],...
    'String','d_g','BackgroundColor',bgcolor);
Con_d_g_Value= uicontrol('Parent',Controller,'Style','text','Position',[750,100,40,25],...
    'String',num2str(d_g),'BackgroundColor',bgcolor);

% beta_AraC
Con_beta_AraC = uicontrol('Parent',Controller,'Style','slider','Position',[650, 125, 100, 25],...
              'value',beta_AraC, 'min',0, 'max',10, 'SliderStep',[0.01 0.10]);
bgcolor = Controller.Color;
Con_beta_AraC_Title= uicontrol('Parent',Controller,'Style','text','Position',[550,125,100,25],...
    'String','beta_AraC','BackgroundColor',bgcolor);
Con_beta_AraC_Value= uicontrol('Parent',Controller,'Style','text','Position',[750,125,40,25],...
    'String',num2str(beta_AraC),'BackgroundColor',bgcolor);
% d_AraC
Con_d_AraC = uicontrol('Parent',Controller,'Style','slider','Position',[650, 150, 100, 25],...
              'value',d_AraC, 'min',0, 'max',1, 'SliderStep',[0.001 0.10]);
bgcolor = Controller.Color;
Con_d_AraC_Title= uicontrol('Parent',Controller,'Style','text','Position',[550,150,100,25],...
    'String','d_AraC','BackgroundColor',bgcolor);
Con_d_AraC_Value= uicontrol('Parent',Controller,'Style','text','Position',[750,150,40,25],...
    'String',num2str(d_AraC),'BackgroundColor',bgcolor);
% K_RA
Con_K_RA = uicontrol('Parent',Controller,'Style','slider','Position',[650, 175, 100, 25],...
              'value',K_RA, 'min',0, 'max',10, 'SliderStep',[0.01 0.10]);
bgcolor = Controller.Color;
```

```
Con_K_RA_Title= uicontrol('Parent',Controller,'Style','text','Position',[550,175,100,25],...
    'String','K_RA','BackgroundColor',bgcolor);
Con_K_RA_Value= uicontrol('Parent',Controller,'Style','text','Position',[750,175,40,25],...
    'String',num2str(K_RA),'BackgroundColor',bgcolor);
% p
Con_p = uicontrol('Parent',Controller,'Style','slider','Position',[650, 200, 100, 25],...
              'value',p, 'min',0, 'max',10, 'SliderStep',[0.05 0.10]);
bgcolor = Controller.Color;
Con_p_Title= uicontrol('Parent',Controller,'Style','text','Position',[550,200,100,25],...
    'String','p','BackgroundColor',bgcolor);
Con_p_Value= uicontrol('Parent',Controller,'Style','text','Position',[750,200,40,25],...
    'String',num2str(p),'BackgroundColor',bgcolor);
% alpha_Acs%%%%%%
Con_alpha_Acs = uicontrol('Parent',Controller,'Style','slider','Position',[650, 225, 100, 25],...
              'value',alpha_ACS, 'min',0, 'max',10, 'SliderStep',[0.01 0.10]);
bgcolor = Controller.Color;
Con_alpha_Acs_Title= uicontrol('Parent',Controller,'Style','text','Position',[550,225,100,25],...
    'String','alpha_Acs','BackgroundColor',bgcolor);
Con_alpha_Acs_Value= uicontrol('Parent',Controller,'Style','text','Position',[750,225,40,25],...
    'String',num2str(alpha_ACS),'BackgroundColor',bgcolor);

% k_Iso
Con_k_Iso = uicontrol('Parent',Controller,'Style','slider','Position',[650, 250, 100, 25],...
              'value',k_Iso, 'min',0, 'max',10, 'SliderStep',[0.01 0.10]);
bgcolor = Controller.Color;
Con_k_Iso_Title= uicontrol('Parent',Controller,'Style','text','Position',[550,250,100,25],...
    'String','k_Iso','BackgroundColor',bgcolor);
Con_k_Iso_Value= uicontrol('Parent',Controller,'Style','text','Position',[750,250,40,25],...
    'String',num2str(k_Iso),'BackgroundColor',bgcolor);

% Fourth Collumn
% Yxs
Con_Yxs = uicontrol('Parent',Controller,'Style','slider','Position',[900, 25, 100, 25],...
              'value',Yxs, 'min',0, 'max',1, 'SliderStep',[0.01 0.10]);
bgcolor = Controller.Color;
Con_Yxs_Title= uicontrol('Parent',Controller,'Style','text','Position',[800,25,100,25],...
    'String','Yxs','BackgroundColor',bgcolor);
Con_Yxs_Value= uicontrol('Parent',Controller,'Style','text','Position',[1000,25,40,25],...
    'String',num2str(Yxs),'BackgroundColor',bgcolor);
% beta_x
Con_beta_x = uicontrol('Parent',Controller,'Style','slider','Position',[900, 50, 100, 25],...
              'value',beta_x, 'min',0, 'max',0.1, 'SliderStep',[0.01 0.10]);
bgcolor = Controller.Color;
Con_beta_x_Title= uicontrol('Parent',Controller,'Style','text','Position',[800,50,100,25],...
    'String','beta_x','BackgroundColor',bgcolor);
Con_beta_x_Value= uicontrol('Parent',Controller,'Style','text','Position',[1000,50,40,25],...
    'String',num2str(beta_x),'BackgroundColor',bgcolor);
% K_Iso
Con_K_Iso = uicontrol('Parent',Controller,'Style','slider','Position',[900, 75, 100, 25],...
              'value',K_Iso, 'min',0, 'max',10, 'SliderStep',[0.01 0.10]);
bgcolor = Controller.Color;
Con_K_Iso_Title= uicontrol('Parent',Controller,'Style','text','Position',[800,75,100,25],...
    'String','K_Iso','BackgroundColor',bgcolor);
```

```
Con_K_Iso_Value= uicontrol('Parent',Controller,'Style','text','Position',[1000,75,40,25],...
    'String',num2str(K_Iso),'BackgroundColor',bgcolor);

% d_Acs
Con_d_Acs = uicontrol('Parent',Controller,'Style','slider','Position',[900, 100, 100, 25],...
              'value',d_ACS, 'min',0, 'max',10, 'SliderStep',[0.01 0.10]);
bgcolor = Controller.Color;
Con_d_Acs_Title= uicontrol('Parent',Controller,'Style','text','Position',[800,100,100,25],...
    'String','d_Acs','BackgroundColor',bgcolor);
Con_d_Acs_Value= uicontrol('Parent',Controller,'Style','text','Position',[1000,100,40,25],...
    'String',num2str(d_ACS),'BackgroundColor',bgcolor);
% beta_AceA
Con_beta_AceA = uicontrol('Parent',Controller,'Style','slider','Position',[900, 125, 100, 25],...
              'value',beta_AceA, 'min',0, 'max',10, 'SliderStep',[0.01 0.10]);
bgcolor = Controller.Color;
Con_beta_AceA_Title= uicontrol('Parent',Controller,'Style','text','Position',[800,125,100,25],...
    'String','beta_AceA','BackgroundColor',bgcolor);
Con_beta_AceA_Value= uicontrol('Parent',Controller,'Style','text','Position',[1000,125,40,25],...
    'String',num2str(beta_AceA),'BackgroundColor',bgcolor);

% d_AceA
Con_d_AceA = uicontrol('Parent',Controller,'Style','slider','Position',[900, 150, 100, 25],...
              'value',d_AceA, 'min',0, 'max',10, 'SliderStep',[0.01 0.10]);
bgcolor = Controller.Color;
Con_d_AceA_Title= uicontrol('Parent',Controller,'Style','text','Position',[800,150,100,25],...
    'String','d_AceA','BackgroundColor',bgcolor);
Con_d_AceA_Value= uicontrol('Parent',Controller,'Style','text','Position',[1000,150,40,25],...
    'String',num2str(d_AceA),'BackgroundColor',bgcolor);
% q
Con_q = uicontrol('Parent',Controller,'Style','slider','Position',[900, 175, 100, 25],...
              'value',q, 'min',0, 'max',10, 'SliderStep',[0.01 0.10]);
bgcolor = Controller.Color;
Con_q_Title= uicontrol('Parent',Controller,'Style','text','Position',[800,175,100,25],...
    'String','q','BackgroundColor',bgcolor);
Con_q_Value= uicontrol('Parent',Controller,'Style','text','Position',[1000,175,40,25],...
    'String',num2str(q),'BackgroundColor',bgcolor);
% k_Ac
Con_k_Ac = uicontrol('Parent',Controller,'Style','slider','Position',[900, 200, 100, 25],...
              'value',k_Ac, 'min',0, 'max',10, 'SliderStep',[0.01 0.10]);
bgcolor = Controller.Color;
Con_k_Ac_Title= uicontrol('Parent',Controller,'Style','text','Position',[800,200,100,25],...
    'String','k_Ac','BackgroundColor',bgcolor);
Con_k_Ac_Value= uicontrol('Parent',Controller,'Style','text','Position',[1000,200,40,25],...
    'String',num2str(k_Ac),'BackgroundColor',bgcolor);
% K_Ac
Con_K_Ac = uicontrol('Parent',Controller,'Style','slider','Position',[900, 225, 100, 25],...
              'value',K_Ac, 'min',0, 'max',10, 'SliderStep',[0.01 0.10]);
bgcolor = Controller.Color;
Con_K_Ac_Title= uicontrol('Parent',Controller,'Style','text','Position',[800,225,100,25],...
    'String','K_Ac','BackgroundColor',bgcolor);
Con_K_Ac_Value= uicontrol('Parent',Controller,'Style','text','Position',[1000,225,40,25],...
    'String',num2str(K_Ac),'BackgroundColor',bgcolor);
% r
```

```
Con_r = uicontrol('Parent',Controller,'Style','slider','Position',[900, 250, 100, 25],...
              'value',r, 'min',0, 'max',10, 'SliderStep',[0.05 0.10]);
bgcolor = Controller.Color;
Con_r_Title= uicontrol('Parent',Controller,'Style','text','Position',[800,250,100,25],...
    'String','r','BackgroundColor',bgcolor);
Con_r_Value= uicontrol('Parent',Controller,'Style','text','Position',[1000,250,40,25],...
    'String',num2str(r),'BackgroundColor',bgcolor);

% Fifth column
% k_aCoA
Con_k_aCoA = uicontrol('Parent',Controller,'Style','slider','Position',[1150, 25, 100, 25],...
              'value',k_aCoA, 'min',0, 'max',10, 'SliderStep',[0.01 0.10]);
bgcolor = Controller.Color;
Con_k_aCoA_Title= uicontrol('Parent',Controller,'Style','text','Position',[1050,25,100,25],...
    'String','k_aCoA','BackgroundColor',bgcolor);
Con_k_aCoA_Value= uicontrol('Parent',Controller,'Style','text','Position',[1250,25,40,25],...
    'String',num2str(k_aCoA),'BackgroundColor',bgcolor);
% alpha_p
Con_alpha_p = uicontrol('Parent',Controller,'Style','slider','Position',[1150, 50, 100, 25],...
              'value',alpha_p, 'min',0, 'max',0.1, 'SliderStep',[0.01 0.10]);
bgcolor = Controller.Color;
Con_alpha_p_Title= uicontrol('Parent',Controller,'Style','text','Position',[1050,50,100,25],...
    'String','alpha_p','BackgroundColor',bgcolor);
Con_alpha_p_Value= uicontrol('Parent',Controller,'Style','text','Position',[1250,50,40,25],...
    'String',num2str(alpha_p),'BackgroundColor',bgcolor);
% beta_p
Con_beta_p = uicontrol('Parent',Controller,'Style','slider','Position',[1150, 75, 100, 25],...
              'value',beta_p, 'min',0, 'max',0.01, 'SliderStep',[0.01 0.10]);
bgcolor = Controller.Color;
Con_beta_p_Title= uicontrol('Parent',Controller,'Style','text','Position',[1050,75,100,25],...
    'String','beta_p','BackgroundColor',bgcolor);
Con_beta_p_Value= uicontrol('Parent',Controller,'Style','text','Position',[1250,75,40,25],...
    'String',num2str(beta_p),'BackgroundColor',bgcolor);
% K_PaCoA
Con_K_PaCoA = uicontrol('Parent',Controller,'Style','slider','Position',[1150, 100, 100, 25],...
              'value',K_PaCoA, 'min',0, 'max',100, 'SliderStep',[0.01 0.10]);
bgcolor = Controller.Color;
Con_K_PaCoA_Title= uicontrol('Parent',Controller,'Style','text','Position',[1050,100,100,25],...
    'String','K_PaCoA','BackgroundColor',bgcolor);
Con_K_PaCoA_Value= uicontrol('Parent',Controller,'Style','text','Position',[1250,100,40,25],...
    'String',num2str(K_PaCoA),'BackgroundColor',bgcolor);
% v
Con_v = uicontrol('Parent',Controller,'Style','slider','Position',[1150, 125, 100, 25],...
              'value',v, 'min',0, 'max',10, 'SliderStep',[0.05 0.10]);
bgcolor = Controller.Color;
Con_v_Title= uicontrol('Parent',Controller,'Style','text','Position',[1050,125,100,25],...
    'String','v','BackgroundColor',bgcolor);
Con_v_Value= uicontrol('Parent',Controller,'Style','text','Position',[1250,125,40,25],...
    'String',num2str(v),'BackgroundColor',bgcolor);
% Yps
Con_Yps = uicontrol('Parent',Controller,'Style','slider','Position',[1150, 150, 100, 25],...
              'value',Yps, 'min',0, 'max',1, 'SliderStep',[0.01 0.10]);
bgcolor = Controller.Color;
```

```
Con_Yps_Title= uicontrol('Parent',Controller,'Style','text','Position',[1050,150,100,25],...
    'String','Yps','BackgroundColor',bgcolor);
Con_Yps_Value= uicontrol('Parent',Controller,'Style','text','Position',[1250,150,40,25],...
    'String',num2str(Yps),'BackgroundColor',bgcolor);
% k_c
Con_k_c = uicontrol('Parent',Controller,'Style','slider','Position',[1150, 175, 100, 25],...
              'value',k_c, 'min',0, 'max',10, 'SliderStep',[0.01 0.10]);
bgcolor = Controller.Color;
Con_k_c_Title= uicontrol('Parent',Controller,'Style','text','Position',[1050,175,100,25],...
    'String','k_c','BackgroundColor',bgcolor);
Con_k_c_Value= uicontrol('Parent',Controller,'Style','text','Position',[1250,175,40,25],...
    'String',num2str(k_c),'BackgroundColor',bgcolor);
% s
Con_s = uicontrol('Parent',Controller,'Style','slider','Position',[1150,200, 100, 25],...
              'value',s, 'min',0, 'max',10, 'SliderStep',[0.05 0.10]);
bgcolor = Controller.Color;
Con_s_Title= uicontrol('Parent',Controller,'Style','text','Position',[1050,200,100,25],...
    'String','s','BackgroundColor',bgcolor);
Con_s_Value= uicontrol('Parent',Controller,'Style','text','Position',[1250,200,40,25],...
    'String',num2str(s),'BackgroundColor',bgcolor);


% Now each controller button needs a listener.

addlistener(Con_mu_max, 'Value', 'PostSet', @callbackfn);
addlistener(Con_w, 'Value', 'PostSet', @callbackfn);
addlistener(Con_K_m, 'Value', 'PostSet', @callbackfn);
addlistener(Con_K_1, 'Value', 'PostSet', @callbackfn);
addlistener(Con_K_2, 'Value', 'PostSet', @callbackfn);
addlistener(Con_k_1, 'Value', 'PostSet', @callbackfn);
addlistener(Con_k_2, 'Value', 'PostSet', @callbackfn);
addlistener(Con_n, 'Value', 'PostSet', @callbackfn);
addlistener(Con_m, 'Value', 'PostSet', @callbackfn);
addlistener(Con_alpha_g, 'Value', 'PostSet', @callbackfn);
addlistener(Con_d_g, 'Value', 'PostSet', @callbackfn);
addlistener(Con_beta_AraC, 'Value', 'PostSet', @callbackfn);
addlistener(Con_K_RA, 'Value', 'PostSet', @callbackfn);
addlistener(Con_d_AraC, 'Value', 'PostSet', @callbackfn);
addlistener(Con_p, 'Value', 'PostSet', @callbackfn);
addlistener(Con_alpha_Acs, 'Value', 'PostSet', @callbackfn);
addlistener(Con_d_Acs, 'Value', 'PostSet', @callbackfn);
addlistener(Con_beta_AceA, 'Value', 'PostSet', @callbackfn);
addlistener(Con_K_RAraC, 'Value', 'PostSet', @callbackfn);
addlistener(Con_d_AceA, 'Value', 'PostSet', @callbackfn);
addlistener(Con_q, 'Value', 'PostSet', @callbackfn);
addlistener(Con_k_Ac, 'Value', 'PostSet', @callbackfn);
addlistener(Con_K_Ac, 'Value', 'PostSet', @callbackfn);
addlistener(Con_r, 'Value', 'PostSet', @callbackfn);
addlistener(Con_k_aCoA, 'Value', 'PostSet', @callbackfn);
addlistener(Con_alpha_p, 'Value', 'PostSet', @callbackfn);
addlistener(Con_beta_p, 'Value', 'PostSet', @callbackfn);
addlistener(Con_K_PaCoA, 'Value', 'PostSet', @callbackfn);
addlistener(Con_v, 'Value', 'PostSet', @callbackfn);
```

```
addlistener(Con_Yps, 'Value', 'PostSet', @callbackfn);
addlistener(Con_k_c, 'Value', 'PostSet', @callbackfn);
addlistener(Con_s, 'Value', 'PostSet', @callbackfn);
addlistener(Con_k_Iso, 'Value', 'PostSet', @callbackfn);
addlistener(Con_Yxs, 'Value', 'PostSet', @callbackfn);
addlistener(Con_beta_x, 'Value', 'PostSet', @callbackfn);
addlistener(Con_K_Iso, 'Value', 'PostSet', @callbackfn);
addlistener(Con_A_o, 'Value', 'PostSet', @callbackfn);
addlistener(Con_GDH_o, 'Value', 'PostSet', @callbackfn);
addlistener(Con_AraC_o, 'Value', 'PostSet', @callbackfn);
addlistener(Con_Acs_o, 'Value', 'PostSet', @callbackfn);
addlistener(Con_AceA_o, 'Value', 'PostSet', @callbackfn);
addlistener(Con_Ac_o, 'Value', 'PostSet', @callbackfn);
addlistener(Con_aCoA_o, 'Value', 'PostSet', @callbackfn);
addlistener(Con_WE_o, 'Value', 'PostSet', @callbackfn);
addlistener(Con_C_o, 'Value', 'PostSet', @callbackfn);
addlistener(Con_Iso_o, 'Value', 'PostSet', @callbackfn);
addlistener(Con_S_o, 'Value', 'PostSet', @callbackfn);
addlistener(Con_X_o, 'Value', 'PostSet', @callbackfn);
```

Function to rewrite old variables, resolve ODEs and update plots.

```
    function callbackfn(source, eventdata)
    mu_max = get(Con_mu_max, 'Value');
    w = get(Con_w, 'Value');
    K_m = get(Con_K_m, 'Value');
    K_1 = get(Con_K_1, 'Value');
    K_2 = get(Con_K_2, 'Value');
    k_1 = get(Con_k_1, 'Value');
    k_2 = get(Con_k_2, 'Value');
    n = get(Con_n, 'Value');
    m = get(Con_m, 'Value');
    alpha_g = get(Con_alpha_g, 'Value');
    d_g = get(Con_d_g, 'Value');
    beta_AraC = get(Con_beta_AraC, 'Value');
    d_AraC = get(Con_d_AraC, 'Value');
    K_RAraC = get(Con_K_RAraC, 'Value');
    p = get(Con_p, 'Value');
    alpha_p = get(Con_alpha_p, 'Value');
    alpha_ACS = get(Con_alpha_Acs, 'Value');
    d_ACS = get(Con_d_Acs, 'Value');
    beta_AceA = get(Con_beta_AceA, 'Value');
    K_RA = get(Con_K_RA, 'Value');
    d_AceA = get(Con_d_AceA, 'Value');
    q = get(Con_q, 'Value');
    k_Ac = get(Con_k_Ac, 'Value');
    K_Ac = get(Con_K_Ac, 'Value');
    r = get(Con_r, 'Value');
    k_aCoA = get(Con_k_aCoA, 'Value');
    beta_p = get(Con_beta_p, 'Value');
    K_PaCoA = get(Con_K_PaCoA, 'Value');
    v = get(Con_v, 'Value');
    Yps = get(Con_Yps, 'Value');
```

```
    k_c = get(Con_k_c, 'Value');
    s = get(Con_s, 'Value');
    k_Iso = get(Con_k_Iso, 'Value');
    K_Iso = get(Con_K_Iso, 'Value');
    Yxs = get(Con_Yxs, 'Value');
    beta_x = get(Con_beta_x, 'Value');
    A_o = get(Con_A_o, 'Value');
    GDH_o = get(Con_GDH_o, 'Value');
    AraC_o = get(Con_AraC_o, 'Value');
    Acs_o = get(Con_Acs_o, 'Value');
    AceA_o = get(Con_AceA_o, 'Value');
    AC_o = get(Con_AC_o, 'Value');
    aCoA_o = get(Con_aCoA_o, 'Value');
    WE_o = get(Con_WE_o, 'Value');
    C_o = get(Con_C_o, 'Value');
    Iso_o = get(Con_Iso_o, 'Value');
    S_o = get(Con_S_o, 'Value');
    X_o = get(Con_X_o, 'Value');


    tspan = [1:1:t2];
    initial = [A_o GDH_o AraC_o Acs_o AceA_o AC_o aCoA_o WE_o C_o Iso_o S_o X_o];
    [T,Y]= ode23s(@(t,x) Arabinose_Gui_Functions(t,x, k_1, K_1, n, k_2, K_2, m, alpha_g, d_g,
beta_AraC, K_RA, p, d_AraC, alpha_ACS, d_ACS,beta_AceA, K_RAraC, q, d_AceA, k_AC, K_AC, r,
k_aCoA, k_c, k_Iso, K_Iso, s, mu_max, K_m, w,Yxs, beta_x, alpha_p, beta_p, K_PaCoA, v,
Yps),tspan, initial, options);
    LineA.YData  = Y(:,1);
    LineGDH.YData = Y(:,2);
    LineAraC.YData  = Y(:,3);
    LineACS.YData  = Y(:,4);
    LineAceA.YData  = Y(:,5);
    LineAc.YData  = Y(:,6);
    LineaCoA.YData  = Y(:,7);
    LineWE.YData  = Y(:,8);
    LineC.YData = Y(:,9);
    LineIso.YData  = Y(:,10);
    LineS.YData  = Y(:,11);
    LineX.YData  = Y(:,12);
    PhasePlane.YData  = Y(:,8);
    PhasePlane.XData  = Y(:,12);


    Con_mu_max_Value.String = num2str(mu_max);
    Con_w_Value.String = num2str(w);
    Con_K_m_Value.String = num2str(K_m);
    Con_K_1_Value.String = num2str(K_1);
    Con_K_2_Value.String = num2str(K_2);
    Con_k_1_Value.String = num2str(k_1);
    Con_k_2_Value.String = num2str(k_2);
    Con_n_Value.String = num2str(n);
    Con_m_Value.String = num2str(m);
    Con_alpha_g_Value.String = num2str(alpha_g);
    Con_d_g_Value.String = num2str(d_g);
    Con_beta_AraC_Value.String = num2str(beta_AraC);
```

```
    Con_d_AraC_Value.String = num2str(d_AraC);
    Con_K_RAraC_Value.String = num2str(K_RAraC);
    Con_p_Value.String = num2str(p);
    Con_alpha_Acs_Value.String = num2str(alpha_ACS);
    Con_d_Acs_Value.String = num2str(d_ACS);
    Con_beta_AceA_Value.String = num2str(beta_AceA);
    Con_K_RA_Value.String = num2str(K_RA);
    Con_d_AceA_Value.String = num2str(d_AceA);
    Con_q_Value.String = num2str(q);
    Con_k_Ac_Value.String = num2str(k_Ac);
    Con_K_Ac_Value.String = num2str(K_Ac);
    Con_r_Value.String = num2str(r);
    Con_k_aCoA_Value.String = num2str(k_aCoA);
    Con_alpha_p_Value.String = num2str(alpha_p);
    Con_beta_p_Value.String = num2str(beta_p);
    Con_K_PaCoA_Value.String = num2str(K_PaCoA);
    Con_v_Value.String = num2str(v);
    Con_Yps_Value.String = num2str(Yps);
    Con_k_c_Value.String = num2str(k_c);
    Con_s_Value.String = num2str(s);
    Con_k_Iso_Value.String = num2str(k_Iso);
    Con_Yxs_Value.String = num2str(Yxs);
    Con_beta_x_Value.String = num2str(beta_x);
    Con_K_Iso_Value.String = num2str(K_Iso);
    Con_A_o_Value.String = num2str(A_o);
    Con_GDH_o_Value.String = num2str(GDH_o);
    Con_AraC_o_Value.String = num2str(AraC_o);
    Con_Acs_o_Value.String = num2str(Acs_o);
    Con_AceA_o_Value.String = num2str(AceA_o);
    Con_Ac_o_Value.String = num2str(Ac_o);
    Con_aCoA_o_Value.String = num2str(aCoA_o);
    Con_WE_o_Value.String = num2str(WE_o);
    Con_C_o_Value.String = num2str(C_o);
    Con_Iso_o_Value.String = num2str(Iso_o);
    Con_S_o_Value.String = num2str(S_o);
    Con_X_o_Value.String = num2str(X_o);

    end

  end
```

Published with MATLAB® R2019a
